# Hypovirus infection induces proliferation and perturbs functions of mitochondria in the chestnut blight fungus

**DOI:** 10.3389/fmicb.2023.1206603

**Published:** 2023-06-28

**Authors:** Jinzi Wang, Rui Quan, Xipu He, Qiang Fu, Shigen Tian, Lijiu Zhao, Shuangcai Li, Liming Shi, Ru Li, Baoshan Chen

**Affiliations:** ^1^State Key Laboratory for Conservation and Utilization of Subtropical Agro-Bioresources and College of Life Science and Technology, Guangxi University, Nanning, China; ^2^Guangxi Key Laboratory for Polysaccharide Materials and Modifications, Key Laboratory of Protection and Utilization of Marine Resources, School of Marine Sciences and Biotechnology, Guangxi Minzu University, Nanning, China

**Keywords:** *Cryphonectria parasitica*, hypovirus, mitochondrial proteome, ROS, respiratory efficiency

## Abstract

**Introduction:**

The chestnut blight fungus, *Cryphonectria parasitica*, and hypovirus have been used as a model to probe the mechanism of virulence and regulation of traits important to the host fungus. Previous studies have indicated that mitochondria could be the primary target of the hypovirus.

**Methods:**

In this study, we report a comprehensive and comparative study comprising mitochondrion quantification, reactive oxygen species (ROS) and respiratory efficiency, and quantitative mitochondrial proteomics of the wild-type and virus-infected strains of the chestnut blight fungus.

**Results and discussion:**

Our data show that hypovirus infection increases the total number of mitochondria, lowers the general ROS level, and increases mitochondrial respiratory efficiency. Quantification of mitochondrial proteomes revealed that a set of proteins functioning in energy metabolism and mitochondrial morphogenesis, as well as virulence, were regulated by the virus. In addition, two viral proteins, p29 and p48, were found to co-fractionate with the mitochondrial membrane and matrix. These results suggest that hypovirus perturbs the host mitochondrial functions to result in hypovirulence.

## Introduction

Hypovirus infection results in reduced virulence (hypovirulence) and hypovirulence-associated traits, including reduction of colony pigmentation, suppression of conidiation, and alteration in the expression of a range of genes and protein of its fungal host, in the chestnut blight fungus *Cryphonectria parasitica* (Nuss, 2005). Perturbation of host gene expression (Chun et al., 2020; Aulia et al., 2021), modification of functional proteins (Park et al., 2004; Salamon et al., 2010), protein transportation and translocation (Jo et al., 2019; Ko et al., 2021; Chun et al., 2022), metabolites (Arnone et al., 2002; Dawe et al., 2009), and autophagy (Shi et al., 2019; Li et al., 2022) have been shown to contribute to the altered traits of the host fungus by the virus.

The involvement of mitochondrion in hypovirulence was first implied when a mitovirus CpMV1 was found to locate in the mitochondrion of *C. parasitica* (Polashock and Hillman, 1994; Monteiro-Vitorello et al., 1995). A later study revealed that CpMV1 infection resulted in mitochondrial genome arrangement. Furthermore, mitochondrial mutants with additional mitochondrial elements were found as hypovirulent strains (Baidyaroy et al., 2011a,b; Springer et al., 2013). By comparative transcriptomic analysis, the results of cDNA microarrays demonstrated that the hypovirus CHV1-EP713-infected strain and the mitochondrial hypovirulent strain EP155/*mit2* shared similar gene expression patterns (Allen and Nuss, 2004). These lines of evidence suggested that mitochondrial function is essential for fungal virulence and this organelle is likely the primary target of hypovirus. The fungal host changes in cell structure, oxidative stress, and energy supply were highly consistent between virus and antimicrobial peptides induction, such as Iturin A, which gives great potential for inhibiting and controlling pathogenic fungi mycelium growth and sporulation in the future (Wang et al., 2020). However, the hypoviral impact on mitochondrial function has not been studied intensively at the protein and physiology levels.

In this study, we report the quantitative analysis of mitochondrial proteomes, determination of reactive oxygen species (ROS), respiration rate, and energy production efficiency of both the wild-type virulent strain and the hypovirus-infected hypovirulent strain. It was found that the change of protein profile by hypovirus infection correlated with the alteration of ROS, proliferation of mitochondria, and lowered energy production efficiency.

## Materials and methods

### Strains and growth conditions

The wild-type strain of *C. parasitica* EP155 (ATCC 38755) and its isogenic strain EP155/CHV1-EP713, derived by transfection with full-length ssRNA transcribed from the infectious clone of CHV1-EP713, were cultured and maintained on potato dextrose agar (PDA) medium at room temperature (Chen et al., 1994). For mitochondrial preparation and ROS and respirational efficiency assays, mycelia of 0.25 g were harvested from PDA medium and inoculated into a 500-ml flask containing 200 ml of EP complete liquid medium statically for 2–4 days at room temperature (24–26°C). For detecting the role of ROS in fungal growth and development, ROS inhibitors/scavengers (20 mM N-Acetyl-L-cysteine (NAC) or 20 μM Apocynin, Sigma) were added to the PDA medium. The number of sporation was collected and measured from the PDA medium after 14 days of cultivation.

### Preparation of mitochondria

Fungal mitochondrial samples were extracted and purified following the method described by Meisinger et al. (2000) with modifications. Briefly, fungal mycelia were collected from EP liquid medium and ground into powder in liquid nitrogen. In total, 1 g of powder was mixed with 5 ml of homogenate buffer (600 mM sorbitol, 10 mM Tris pH 7.4, 1 mM EDTA, and 1 mM PMSF) and vortexed with pre-cold glass beads of 1 mm diameter for 8 min on ice. The mixture was centrifuged at 1,500 g for 5 min at 4°C, and then the supernatant was centrifuged at 3,000 *g* for another 5 min to remove insoluble debris. The fungal mitochondria were pelleted by centrifugation at 12,000 *g* for 15 min at 4°C and dissolved in SEM buffer (250 mM sucrose, 1 mM EDTA, 10 mM MOPS, and pH 7.2). The crude mitochondrial preparation could be stored at −80°C or used for sucrose gradient centrifugation immediately.

For purification, an amount of 400 μl of crude mitochondrial preparation was loaded onto a 15% to 60% (w/v) sucrose gradient in EM buffer (10 mM MOPS, pH 7.2, 1 mM EDTA) and subject to ultracentrifugation at 134,000 *g* for 1 h using SW41 rotor (Beckman Coulter, USA). The purified mitochondria were recovered from the interface, diluted with two volumes of SEM buffer, and pelleted by centrifugation at 12,000 *g* for 5 min. The pellet was resuspended in an SEM buffer for electron microscopy or protein labeling for TMT analysis. The concentration of purified mitochondrial proteins was measured using the Bradford method (Bradford, 1976).

### Preparation of mitochondrial total, membrane-associated, and matrix proteins

An amount of 20 μg of sucrose gradient-purified mitochondria was used to extract proteins. The resultant protein samples were dissolved in SEM buffer and kept at −20°C. The membrane and matrix proteins of mitochondria were separated by membrane and cytosol protein extraction kit (Beyotime, China) (Sun et al., 2016; Xiong et al., 2016). For matrix protein extraction, purified mitochondrial preparation was mixed with Buffer A (10% m/v) and kept in an ice bath for 10 min. The mixture was centrifuged at 700 *g* for 10 min at 4°C and the supernatant was collected and further centrifuged at 1, 4,000 g for 30 min at 4°C to remove debris. The mitochondrial matrix protein-containing supernatant was collected and stored at −80°C for future use. The pellet containing membrane protein was vortexed with Buffer B (25% m/v) for 10 min at 4°C and then centrifuged at 14,000 *g* for 5 min at 4°C. The membrane protein-containing supernatant was collected and stored at −80°C for future use.

### Labeling and fractionation of protein peptides

Reduction, alkylation, trypsin digestion, and labeling of mitochondrial samples were performed according to the instruction of the manufacturer (Pierce™ Tandem Mass Tag Reagents, Thermo Scientific, USA). The peptides were labeled with TMT-labeled peptide samples and were pooled and subject to fractionation by using a PolyLC polysulfoethyl aspartamide column (100 mm × 2.1 mm, 5 μm, 300A pore size on Waters 2695 HPLC system) for off-line strong-cation exchange (SCX) chromatography fractionation. A gradient elution of 100% solvent A (10 mM monopotassium phosphate, 15% acetonitrile) to 100% solvent B (500 mM potassium chloride in solvent A) was performed in 40 min at 200 μl/min flow rate.

### LC-MS/MS analysis

The SCX fractions were further separated by reversed-phase high-performance liquid chromatography (RPLC) and the gradient elution was carried out on an RP-C18 column with buffer A (0.1% methanoic acid) and buffer B (0.1% methanoic acid, 84% acetonitrile) (Wang et al., 2018). The mixed peptides were analyzed on an LTQ Orbitrap Velos (Thermo Scientific, USA) using data-dependent mode (MS scan range from m/z 350 to 1,800) with parameters described (Wang et al., 2016). The survey scan was set at 400 m/z with 60,000 mass resolution. Tandem mass spectrometry was carried out with 10 of the most intense precursor ions. MS2 spectrum was acquired in the ion trap analyzer at normal speed. Proteome Discoverer 1.3 (Thermo Scientific, USA) software was used to analyze the raw data with SEQUEST search engine against *C. parasitica* database v2.0 (26 genome scaffolds totaling 43.9 MB, 11,609 gene models) downloaded from JGI website (https://mycocosm.jgi.doe.gov/Crypa2/Crypa2.home.html) (Crouch et al., 2020). Search parameters were set as follows: precursor ion mass tolerance 10 ppm, fragment mass tolerance 0.8 Da, maximum 2 missed cleavages using trypsin as endoprotease, lysine residues as fixed modification, peptide N-termini as variable modification, and false discovery rate (FDR) for maximum 1%.

### Western blot analysis

Protein samples separated in SDS-PAGE gel were transferred onto a PVDF membrane using TE 77 semi-dry transfer unit (GE Healthcare Life Sciences). The blot analysis was carried out using a Pierce ECL Fast Western Blot kit (Thermo Scientific, USA) following the instruction of the manufacturer. The chemiluminescence detection was performed using an ImageQuant LAS 500 system (GE Healthcare Life Sciences).

### ROS and respirational efficiency assays

The mycelium ROS (total ROS) level was measured using a Fungus ROS High-Quality Assay kit (GenMed Scientifics, China) according to the manufacturer's instruction with an excitation wavelength of 490 nm and an absorption wavelength of 530 nm. Mitochondrial ROS (mtROS) level was measured using a Mitochondrial ROS Assay kit (GenMed Scientifics, China). Three biological repeats were performed for each sample.

The mitochondrial respiratory chain complex V (F_0_F_1_-ATP synthase) activity and specific activity assays were measured using Spectroscopy Quantitative Detection kit (GenMed Scientifics) following the manufacturer's instruction with purified mitochondrial preparations. Meanwhile, the mitochondrial membrane potential (MMP) was measured by a JC-1 fluorescence probe (Beyotime, Shanghai, China) (Wang et al., 2020).

### Vesicle preparation and dsRNA extraction

Vesicle samples were prepared according to the previous description (Wang et al., 2013). Fungal mycelia were ground into powder and dissolved with lysis buffer (0.1 M sodium acetate). The purified vesicle pellets were obtained with ultracentrifugation at 360,000 *g* for 90 min. For the detection of viral dsRNA, mitochondrial and vesicle samples equivalent to 20 μg of protein were treated with phenol-chloroform and separated in a 1% agarose gel.

### Real-time quantitative PCR

Total DNA was isolated from fungal mycelium samples as described (Choi and Nuss, 1992). Specific primers from the nuclear genome and mitochondria were designed according to the sequence information of the *C. parasitica* database v2.0. The accumulation levels of relative DNA fragments in the wild-type strain EP155 and hypovirus-infected strain EP155/CHV1-EP713 were determined by real-time quantitative PCR (qPCR) as described previously (Lan et al., 2008). The PCR was performed with a SuperReal PreMix kit (TIANGEN, China) using LightCycler 480II (Roche, Switzerland).

### Localization/visualization of viral proteins

The cDNA sequences of viral p29 and p48 were inserted into the pCPXHY2 vector with a green fluorescent protein (GFP) tag. The plasmids were transformed into EP155 protoplast and the transformed strains selected from the regeneration medium were screened for antibiotic resistance for three rounds and further purified by single spore isolation. GFP expression was detected by fluorescence microscopy (Olympus Life Science, BX41).

### Transmission electron microscopy

For *in situ* mitochondria analysis, fungal hyphae were cultured in EP complete liquid medium statically for 3 days and scraped and fixed with 2.5% glutaraldehyde in 100 mM phosphate buffer (pH 7.2) at 4°C overnight. After rinsing with phosphate buffer (50 mM, pH 6.8), the samples were dehydrated and embedded. The ultrathin sections were stained in uranium acetate (2%) and post-stained with lead citrate (Shi et al., 2019). The observation was performed on a Hitachi H-7650 transmission electron microscope (Hitachi, Japan) at 80 kV. Three biological repeats were performed for each sample.

## Results and discussion

### Hypovirus infection increases mitochondrial yield

**Figure 1:**
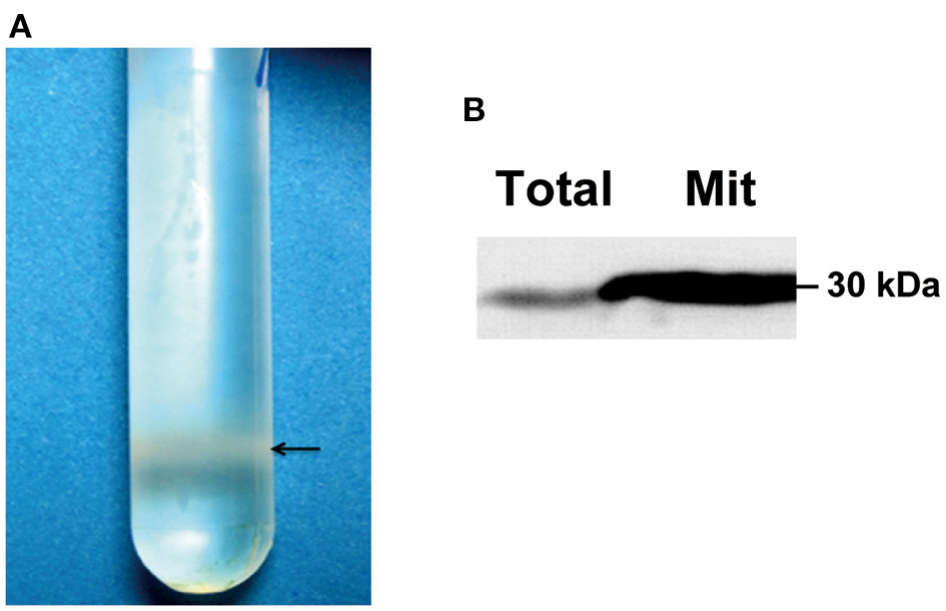
Quality validation of purified fungal mitochondria. **(A)** Purification of mitochondria by sucrose gradient centrifugation. An amount of 400 μl of crude mitochondrial preparation from EP155 was loaded onto a 15% to 60% (w/v) sucrose gradient and ultracentrifuged at 134,000 *g* for 1 h. The purified mitochondria were concentrated at the position near the bottom of the gradient. **(B)** Evaluation of mitochondrion quality by Western blotting. An amount of 20 μg of fungal total and mitochondrial proteins from EP155 was used for Western blot analysis using the ECL method. Prohibitin antibody was used to probe the mitochondrial-specific marker, prohibitin.

Compared with single-cell yeast, the hypha of a filamentous fungus is much more difficult to lyse by enzymatic digestion. To prepare a sufficient amount of mycelia for proteomic analysis, we used the liquid nitrogen-grinding method to homogenize the mycelium and purified the mitochondria by sucrose density gradient centrifugation. As shown in Figure 1A, the mitochondria concentrated at the lower part of the sucrose gradient after centrifugation. Mitochondrial-specific marker prohibitin was highly enriched in the purified mitochondrial protein sample as compared with the total protein sample detected by Western blotting (Figure 1B).

**Figure 2:**
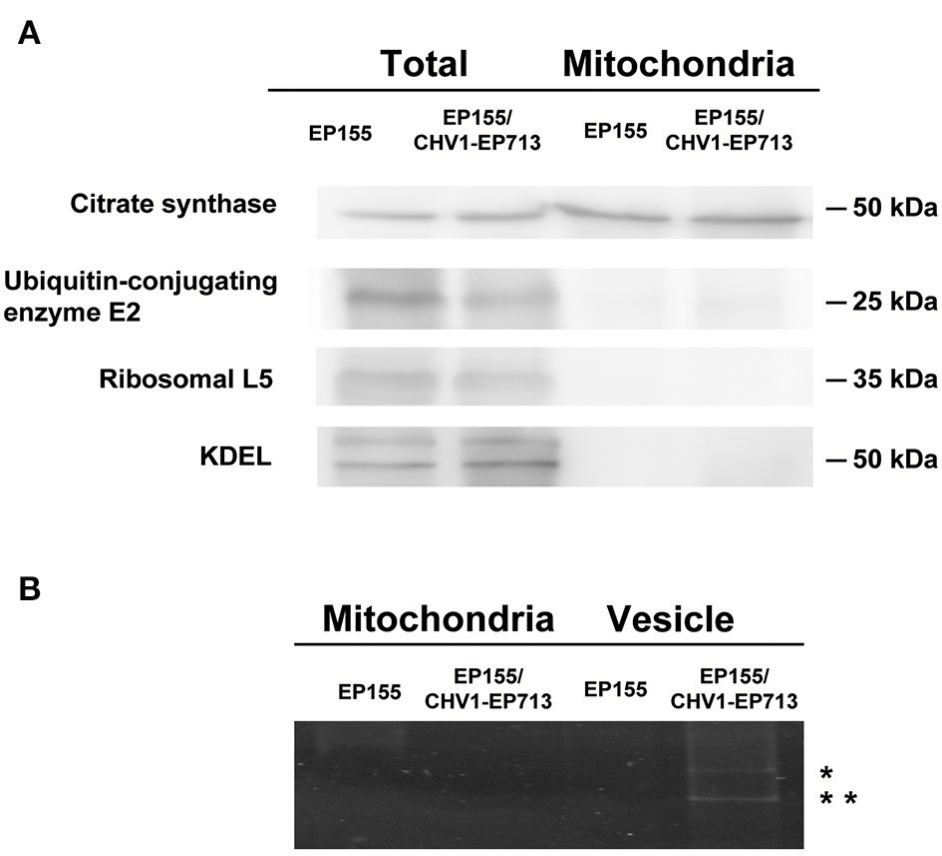
Western blot analysis and RNA electrophoresis of purified fungal mitochondria. **(A)** Detection of marker proteins in total fungal and mitochondria proteins. An amount of 20 μg of protein for each strain was used. After the separation in SDS–PAGE, the proteins were transferred to the PVDF membrane. Antibodies were purchased from Bioss company (anti-citrate synthetase antibody, bs-17709R; anti-ubiquitin-conjugating enzyme E2 antibody, bs-8379R; anti-ribosomal L5 antibody, bs-6573R; anti-KDEL antibody, bs-6940R). **(B)** DsRNA detection of mitochondria and vesicle samples. An amount of 20 μg of protein equivalent from each sample was treated with phenol-chloroform and separated in 1% agarose gel by electrophoresis. dsRNA bands were only observed in virus-infected fungal vesicles (indicated with *).

To rule out the possibility of contamination, the mitochondrial preparation was further assayed by Western blotting with antibodies specific to the mitochondrion (citrate synthase) and to other organelles (cytoplasmic markers ubiquitin-conjugating enzyme E2 and ribosomal L5, and endoplasmic reticulum marker lys-asp-glu-leu KDEL). As shown in Figure 2A, citrate synthase was enriched in the mitochondrial preparation but not the non-mitochondrial proteins. We also tested the hypoviral dsRNAs that are associated with the vesicles in hypovirus-infected strains using the previously described methods (Wang et al., 2013). Meanwhile, no sign of dsRNA was detected in the mitochondrial sample (Figure 2B), confirming that the mitochondria prepared were free of contamination with other cell components.

We obtained, per gram of fresh mycelium, an average yield of 100 μg of a mitochondrion from virus-free EP155 and 500 μg of a mitochondrion from virus-infected EP155/CHV1-EP713 in three independent extractions. The concentration detection of average mitochondrial proteins indicated the increased mitochondrial ratio upon hypovirus infection. According to viral peptide abundance information from mass spectrometry data and mitochondrial protein abundance map from two-dimensional fluorescence difference gel electrophoresis (unpublished data), the abundance of co-purified viral proteins with the mitochondria was <1% of total mitochondrial proteins, which has a negligible effect on the concentration measurement.

DNA quantification of three mitochondrial gene fragments by the qPCR method was carried out to further verify the increased number of mitochondria upon hypovirus infection. The mitochondrial gene, Cytochrome C oxidase subunit IV (COX4), was chosen as a reference (*C. parasitica* database v2.0, located scaffold_4: 2649864-2651087). As shown in Table 1, mitochondrial DNA in hypovirus-infected EP155/CHV1-EP713 was 4.3- to 5.8-folds of that in virus-free EP155.

**Table 1:** Comparative mitochondrial qPCR analysis of EP155 and EP155/CHV1-EP713.

Gene ID^*^	**Primers**	**Primers' position in mitochondrial assembly**	**Amplification product length**	**Amplification ratio. EP155/CHV1-EP713/EP155**	**Reference gene**
Mit 1	5′AGGTGTTCTAAATTTACCATGC3′	Crypa2|mitochondria.fasta:294-315	135 bp	5.8	Cox4
	5′GCTTTTATCCAGTCTGAATT3′	Crypa2|mitochondria.fasta:409-428			
Mit 2	5′ATAATAATGCAACCTTTGGG3′	Crypa2|mitochondria.fasta:13757-13776	130 bp	5.0	Cox4
	5′GACCACATTGGGAATGAAAA3′	Crypa2|mitochondria.fasta:13867-13886			
Mit 3	5′TCCCCGTCTACTACTTGATA3′	Crypa2|mitochondria.fasta:20729-20748	144 bp	4.3	Cox4
	5′CTTGCAGATAATAAAGGACA3′	Crypa2|mitochondria.fasta:20853-20872			

^*^Mitochondrial genome sequence was from C. parasitica database v2.0 mitochondrial assembly.

**Figure 3:**
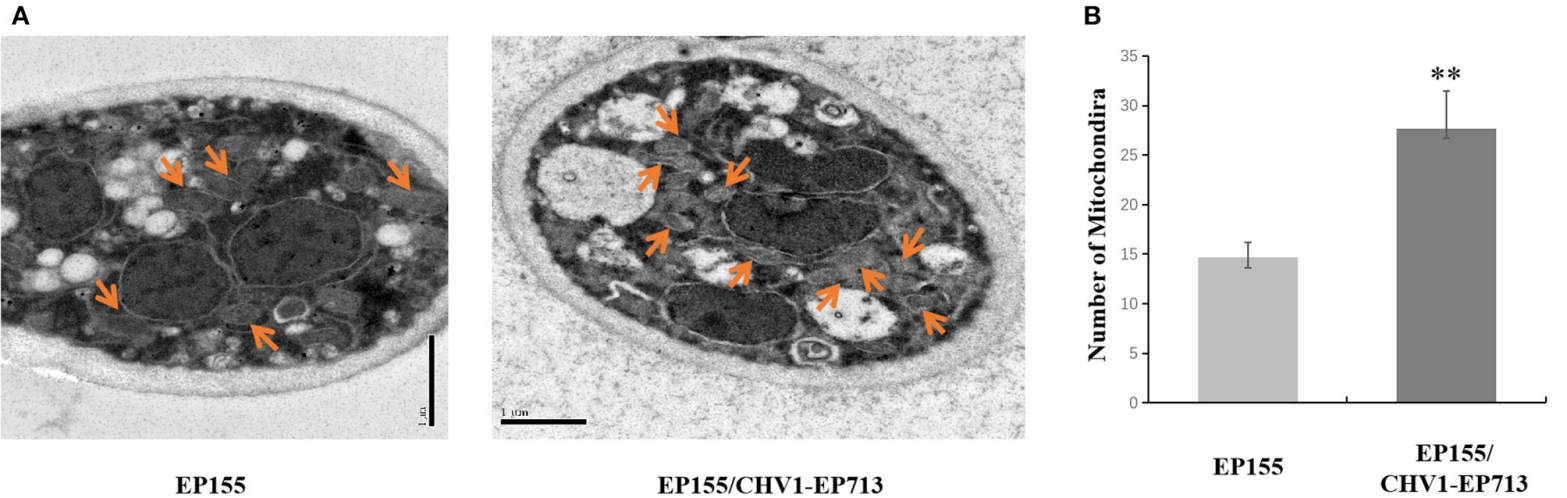
Transmission electron micrographs of *in situ* mitochondria of the fungal cell. **(A)** The intracellular structure of EP155/CHV1-EP713 showed much more membrane structural units compared with EP155. Arrow indicates fungal mitochondria. **(B)** The number of fungal mitochondria through sample repetition and manual counting. There is a significant difference in mitochondrial quantity by statistical analysis (** indicates *P* < 0.01, determined by Student's *t*-test).

To verify the unexpected increase of mitochondrion in the hypovirus-infected strain, we then used transmission electron microscopy (TEM) to compare the ultrastructure of the virus-free EP155 and hypovirus-infected EP155/CHV1-EP713 strains. More mitochondria and membrane structures and abnormal nucleus with large cavitation areas were present in EP155/CHV1-EP713, as compared with EP155 (Figure 3).

### Hypovirus infection reduces ROS levels but elevates mitochondrial respiration

The unexpected increase in mitochondrion number in the hypovirus-infected strain prompted us to measure the mitochondrial activity. To check whether the viral infection would trigger ROS response in the fungus, we measured the total ROS and mitochondrial ROS (mtROS) in the cell of *C. parasitica* at three-time points: day 2, day 3, and day 4. The results showed that both total ROS and mtROS levels were lowered in the hypovirus-infected strain on days 2 and 3, but increased gradually on day 4, while the respiratory efficiency of the virus-infected strain was much higher (440%, 272%) on days 2 and 3 than the virus-free strain, but reduced sharply on day 4 (65%) as compared with the virus-free strain EP155 (Table 2).

**Table 2:** ROS level and respiration efficiency of *C. parasitica* in EP155 and EP155/CHV1-EP713.

**Strains time point**	**Total ROS^a^**	**MtROS^b^**	**Respiration efficiency of complex V^c^**
EP155 Day 2	542 ± 11	882 ± 42	0.049 ± 0.013
EP155 Day 3	557 ± 38	405 ± 28	0.032 ± 0.007
EP155 Day 4	297 ± 15	412 ± 16	0.195 ± 0.012
EP155/CHV1-EP713 Day 2	464 ± 38	726 ± 23	0.216 ± 0.015
EP155/CHV1-EP713 Day 3	336 ± 22	320 ± 19	0.087 ± 0.009
EP155/CHV1-EP713 Day 4	304 ± 18	372 ± 35	0.127 ± 0.018

^a^The average numbers represent fluorescence values from 100 mg mycelium with 3 times biological repeats with standard deviation.

^b^The average numbers represent fluorescence value from 50 μg purified mitochondrion with 3 times biological repeats with standard deviation.

^c^The average numbers of micromole NADH/min/milligram mitochondria with 3 times biological repeats with standard deviation.

**Figure 4:**
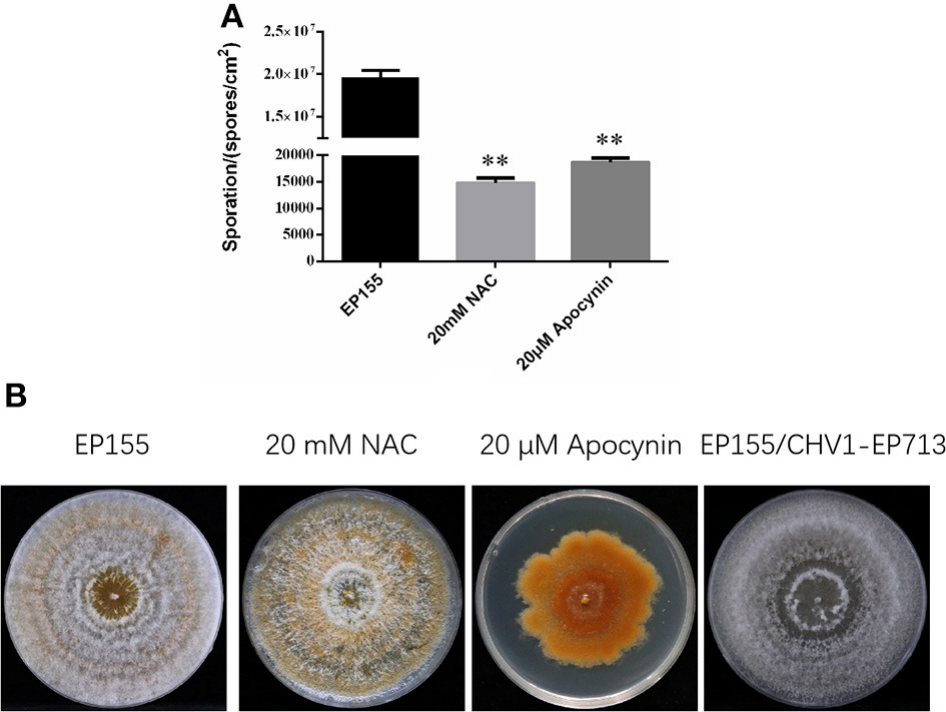
The experiment of ROS inhibitors/scavengers. **(A)** The decreased sporation of EP155 after being treated with 20 mM NAC and 20 μM Apocynin, respectively. Values were calculated from three biological repeats. Bars indicate mean deviations. ** indicates *P* < 0.01, determined by Student's *t*-test. **(B)** The phenotype of EP155 after being treated with 20 mM NAC and 20 μM Apocynin, respectively.

The reactive oxygen species plays an important role in fungal development and pathogenesis including hyphal growth, conidial differentiation, fruiting body, and apoptosis induction. Furthermore, virus infection and dysfunctional mitochondria often induce ROS levels into chaos. The abnormal ROS level may result in mitochondrial energy collapse and reduce fungal pathogenicity in return (Tudzynski et al., 2012; Segal and Wilson, 2018). The previous study proved that viral protein p29 changed fungal phenotypes as a symptom determinant (Craven et al., 1993). The experimental results of ROS inhibitors/scavengers also showed similar phenotypic changes in part (Figure 4), suggesting that the key metabolic features are associated with hypovirus infection. The decrease in ROS was likely to influence fungal development, such as conidial production and pigment accumulation, and reduce virulence directly or indirectly.

**Figure 5:**
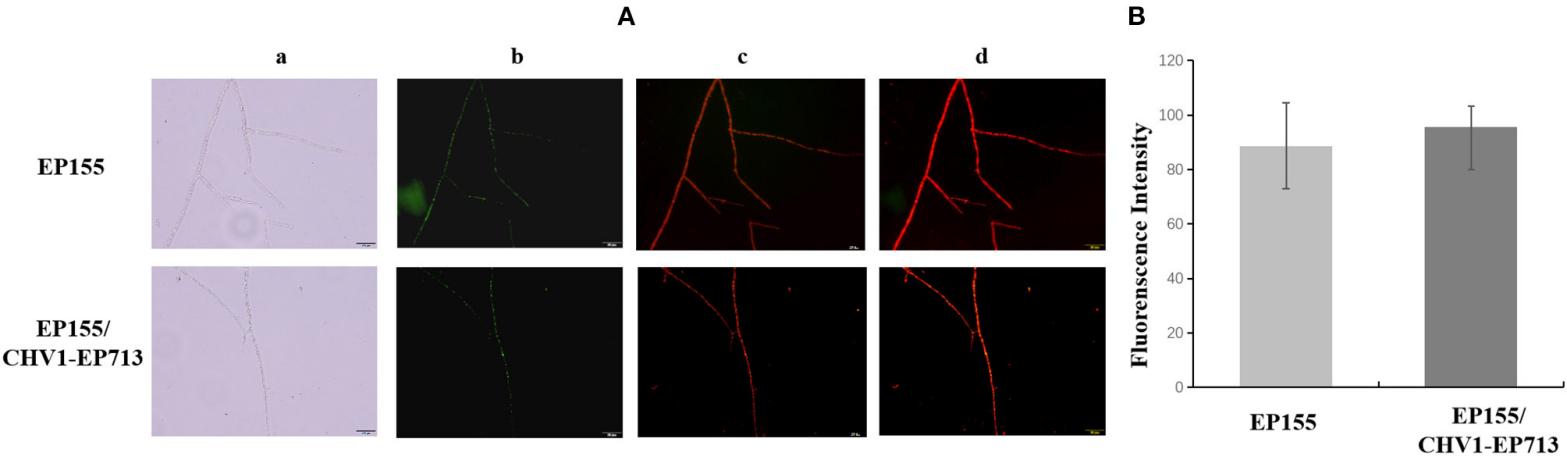
Effect of hypovirus infection on the mitochondrial membrane potential (MMP) of fungal mycelium. **(A)** Label result of JC-1 fluorescent probe. a: Optical channel, b: Green fluorescence channel, c: Red fluorescence channel, d: Red and green channels merged. **(B)** The merged fluorescence value of fungal mycelium from EP155 and EP155/CHV1-EP713. No significant change of MMP was found upon hypovirus infection.

It was reported that treatment of the potato late blight pathogen *Phytophthora infestans* with IturinA extracted from *Bacillus subtilis* WL-2 altered the mitochondrial membrane potential (MMP) (Wang et al., 2020). We determined the MMP of EP155 and EP155/CHV1-EP713 by the JC-1 method. As shown in Figure 5, there were no significant changes in MMP between the two strains, suggesting that virus infection may not disturb the enzyme activity of the respiratory chain, which is in charge of energy production in the cell.

By taking into account the number of mitochondria in each cell, respiratory efficiency for a single mitochondrion was about the same for both strains on day 2, decreased by half in the hypovirus-infected strain on day 3, and 13% on day 4 (Table 2). Changes in mitochondrial size and number had been reported in a mitovirus-infected fungus before (Park et al., 2006). It is likely that the virus replication and transport in host cells would need additional energy, and thus cell resources were hijacked to proliferate more mitochondria for energy generation since the increase in energy production could elevate the mtROS level (Rhoads et al., 2006). On the other hand, the viral infection seems to accelerate the senescence of a mitochondrion.

### Hypovirus infection alters protein patterns in mitochondria

LC-MS/MS analysis of TMT-labeled mitochondrial protein preparations identified a total of 723 proteins (Supplementary Table 1). Among these, 69 proteins were differentially accumulated upon hypovirus infection, of which 33 were upregulated and 36 were downregulated (Table 3). The differentially expressed proteins were compared with the cDNA microarray analysis. Despite the different expression levels and culture conditions, the virus-induced stimulation to mitochondria through the upregulated cytochrome was significant and consistent (Allen and Nuss, 2004; Park et al., 2006; Chun et al., 2020). These differentially accumulated proteins are mainly involved in metabolism, energy production, transport, signal transduction, mitochondrial morphology, stress response, and virulence (Myasoedova, 2008; Ye et al., 2014), suggesting that hypovirus infection disturbs the broad spectrum of the function of the host mitochondrion. Screening of the proteins by UniProtKB GO-cellular component combined with protein function annotation identified 439 proteins (c. 60%) to be located in mitochondria. Furthermore, 68 proteins (c. 10%) were identified with unknown locations, and the remaining 30% of proteins were known to localize outside mitochondria. The situation was very similar with yeast mitochondrial proteome (Prokisch et al., 2004). It is not clear why these proteins appeared in mitochondrial preparations, either by contamination, physical interaction with other cellular compartments, or multiple localizations of existent proteins. Nevertheless, proteome analyses confirmed that the mitochondrion prepared in our study was reasonably pure and thus reliable.

**Table 3:** Differentially expressed mitochondrial proteins upon hypovirus infection.

**Protein ID^a^**	**Protein name**	**Average change (EP713/EP155)^b^**	**Unique PepCount^c^**	**Cover percent^d^**	**MW^e^**	**pI^f^**
**Carbohydrate metabolism**						
266329	2-oxoisovalerate dehydrogenase subunit beta	2.0	1	2.1	41	6.7
346917	Citrate synthase	1.8	5	11.8	52	7.7
100278	Oligosaccharyl transferase STT3 subunit	1.8	1	2.4	83	8.2
342893	Dolichyl-diphosphooligosaccharide-protein (Oligosaccharyltransferase, gamma subunit)	1.6	1	2.4	37	8.7
323583	6-phosphogluconate dehydrogenase, decarboxylase	0.7	9	17.1	54	5.9
293608	Alpha-1,3-glucan synthase	0.6	1	0.5	263	5.8
104818	Glycoside hydrolase family 72 protein	0.6	1	2.6	57	4.3
101684	Glyceraldehyde-3-phosphate dehydrogenase	0.5	13	36.5	36	7.0
**Amino acid metabolism**						
277618	Proline dehydrogenase	3.0	4	9.4	48	8.4
347121	Cyanide hydratase	2.7	3	8.1	40	5.5
98901	Delta-1-pyrroline-5-carboxylate dehydrogenase	2.2	20	39.3	63	8.3
297033	Oxidoreductase (Kynurenine 3-monooxygenase)	1.8	2	7.2	49	6.7
355226	S-adenosylmethionine synthase	0.7	4	12.1	43	6.3
106743	Carbamoyl-phosphate synthase subunit	0.6	5	12.9	50	7.2
99282	Asparagine synthetase	0.5	2	3.1	66	6.4
320111	Carboxypeptidase y protein	0.4	1	1.5	61	4.9
**Lipid metabolism**						
344593	PAP2-domain-containing protein	2.0	2	6.3	37	8.5
280920	Delta fatty acid desaturase protein	0.6	1	2.4	55	8.0
275917	Acyl-coenzyme A oxidase	0.5	2	4.3	78	8.8
321389	3-ketoacyl-thiolase protein	0.5	1	2.2	44	6.3
322936	Cyclopentanol dehydrogenase	0.3	2	6.7	30	6.4
**Energy metabolism**						
337351	Cytochrome c peroxidase	2.2	6	23.5	42	7.9
358627	Short-chain dehydrogenase	2.2	5	16.2	38	8.9
348284	Cytochrome P450	1.9	3	3.5	106	7.2
248835	Monooxygenase FAD-binding protein	1.8	1	2.3	48	6.4
343596	Oxidoreductase	1.7	1	2.3	46	8.5
323634	Thioredoxin protein	1.6	2	6.8	37	4.9
285853	Formate dehydrogenase	1.5	9	24.3	49	8.5
339855	Oxidoreductase domain containing protein	1.5	3	8.7	46	7.9
322591	Flavocytochrome c	0.6	13	20.7	68	6.7
295721	NADH:flavin oxidoreductase NADH oxidase	0.6	2	4.9	44	5.9
357133	NADP-dependent leukotriene B4 12-hydroxydehyd	0.5	2	7.1	31	6.9
355099	Inorganic pyrophosphatase	0.5	5	18.4	33	5.6
357360	Mitochondrial peroxiredoxin prx1	0.5	6	22.7	26	6.2
253099	NADH:flavin oxidoreductase/NADH oxidase	0.3	3	8.7	50	6.4
**Transport**						
356670	Mitochondrial phosphate carrier protein	2.7	3	6.8	42	9.2
248376	Integral membrane protein	1.9	1	5.4	19	9.1
102464	ADP-ribosylation factor	1.7	2	9.8	21	6.6
355657	Rab GDP-dissociation inhibitor	1.7	5	11.7	51	5.9
74450	Mitochondrial Rho GTPase	1.5	2	3.2	70	6.0
322230	Methyltransferase	1.5	2	12.1	30	4.9
96221	BAR-domain-containing protein	0.6	1	4.6	30	6.4
273042	RHO3 protein	0.6	1	4.3	23	4.8
67026	GNAT family acetyltransferase	0.4	1	5.5	24	6.6
358399	N-acetyltransferase-like protein	0.4	2	8.8	31	5.9
245698	3-oxoacyl-[acyl-carrier-protein] reductase	0.3	2	2.2	83	8.5
102797	Serine-type endopeptidase-like protein	0.2	3	6.5	55	5.6
**Signal transduction**						
285804	Alkaline phosphatase family protein	2.0	2	3.3	68	5.8
281836	Telomere silencing protein	0.7	1	1.2	98	6.7
66486	Serine/threonine-protein kinase 12	0.6	1	1.6	104	9.1
336942	Acyl-coenzyme A oxidase	0.5	1	1.2	83	6.7
108039	Serine threonine kinase irei protein	0.4	1	0.7	131	6.3
**Mitochondrial morphology**						
100977	Importin subunit alpha	1.5	8	14.6	60	5.1
344061	VTS1 protein	0.7	1	1.3	65	8.6
320360	60S ribosomal protein L8	0.6	1	3.5	28	11.0
292762	RNA interference and gene silencing protein	0.4	2	2.3	103	9.2
**Others**						
323083	Lea domain-containing protein	1.6	1	8.0	13	9.8
292269	Hrf1 domain-containing protein	1.5	1	3.8	36	9.7
102543	Sphingolipid long chain base-responsive protein	0.6	4	11.2	40	5.6
104580	Transcription factor	0.6	1	4.2	36	8.7
**Unknown**						
350456	Uncharacterized protein	1.7	1	1.5	43	5.2
337183	Uncharacterized protein	2.7	3	6.8	45	9.1
321541	Uncharacterized protein	2.0	4	37.2	13	11.3
356299	Uncharacterized protein	11.3	1	15.1	11	11.3
330389	Uncharacterized protein	7.5	2	6.0	60	6.8
258562	Uncharacterized protein	1.9	6	28.1	31	9.9
340397	Uncharacterized protein	0.6	1	2.4	56	6.6
247407	Uncharacterized protein	0.6	1	8.2	10	5.1
346932	Uncharacterized protein	0.6	1	2.7	53	8.0

The list of up/downregulated mitochondrial protein upon hypovirus infection by HPLC-ESI-OrbiTrap MSMS.

^a^Accession number from Cryphonectria parasitica database v2.0.

^b^Hypovirus-free strain EP155 was set as reference. The average change (EP713/EP155) were from three independent experimental data.

^c^The total number of unique matched peptides to the identified protein.

^d^The cover percentage of matched peptides to the protein sequence.

^e^Theoretical molecular weight (kDa).

^f^Theoretical pI.

**Figure 6:**
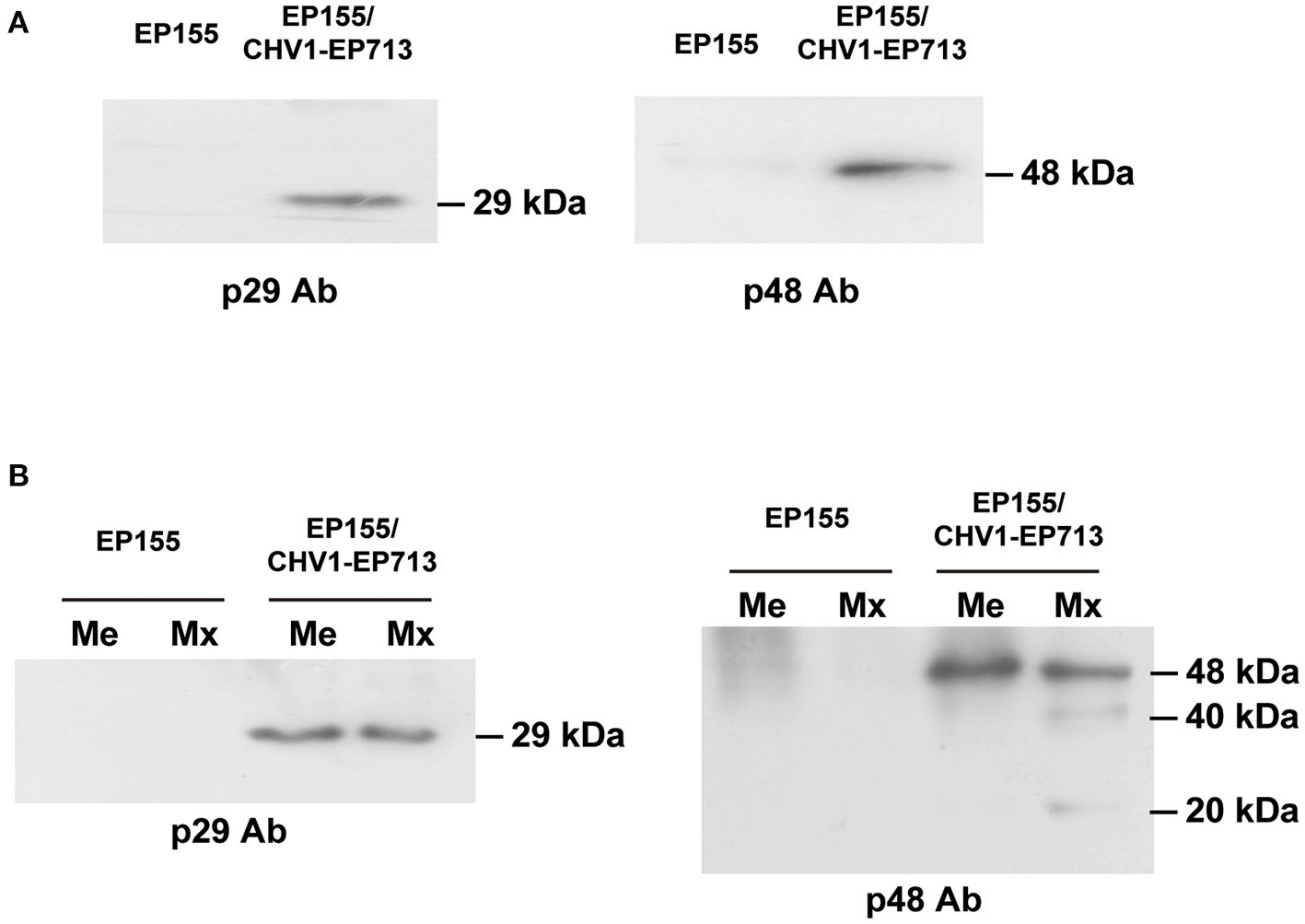
Western blot analysis of virus-encoded proteins in mitochondria. **(A)** Detection of viral proteins p29 and p48 in fungal mitochondrial proteins. An amount of 20 μg of mitochondrial protein for each strain was used. Viral proteins p29 and p48 were only detected in the mitochondria of hypovirus-infected strain EP155/CHV1-EP713. **(B)** Detection of isoforms of viral proteins in fungal mitochondria. The fungal mitochondrial proteins were separated into membrane and matrix fractions. Viral proteins were detected in both fractions in the hypovirus-infected strain but isoforms of p48 were found to exist only in the matrix fraction.

Among the identified peptide information, a peptide (SIGLSHEAAVELVR) was observed from the mitochondria of the hypovirus-infected strain (Supplementary Table 1). This peptide belongs to a 48 kDa viral protein, p48, processed from the viral ORFB polyprotein. For confirmation of viral proteins in fungal mitochondria, Western blot screenings were performed using antibodies against viral proteins p29, p40, and p48. Only p29 and p48 were detected in EP155/CHV1-EP713 (Figure 6A). The viral protein p29 is a papain-like protease, similar to potyvirus HC-Pro protease (Suzuki et al., 1999). p29 was not identified by mass spectrometry but detected by Western blot analysis, likely due to its hydrophobic nature (Wang et al., 2013). We further confirmed that both proteins were present in membrane and matrix fractions of the mitochondrion. It was noticed that isoforms of p48 were predominantly present in the matrix (Figure 6B), suggesting a processing event may have occurred during transmembrane transport or within the matrix.

From the proteomic data, the process of membrane transport of the viral proteins was difficult to explain without details of the related gene function study between the hypovirus and the host cell. However, viral protein PB1-F2 of influenza A was confirmed to translocate into mitochondria *via* Tom (the translocase of the outer mitochondrial membrane) channels in a previous study (Yoshizumi et al., 2014). The further study of hypovirus-induced changes in mitochondrial bioenergetics will bring clues for predicting and understanding possible transport mechanisms of hypovirus proteins. Since a similar modification of viral proteins also existed in transport vesicles (Wang et al., 2013), we suspect that the isoforms of viral p48 may have an impact on the fungal cell, yet details have to be uncovered.

**Figure 7:**
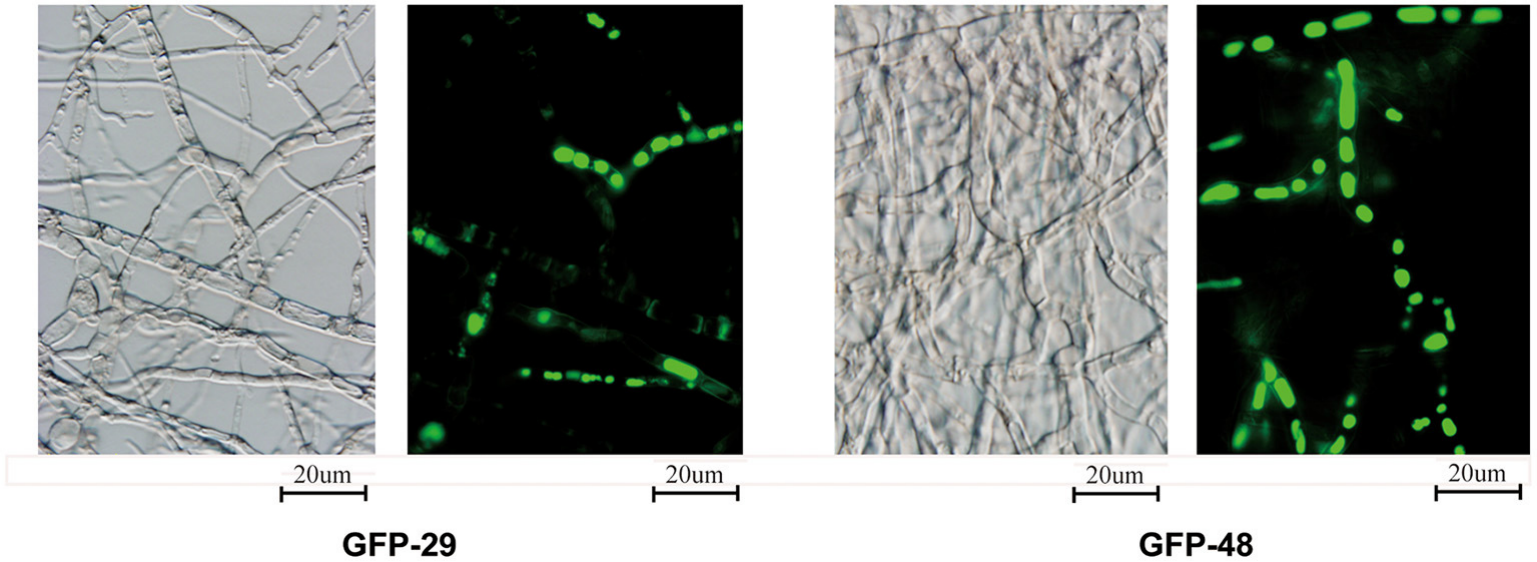
Localization of viral proteins fused with GFP. The transformed strains from EP155 were inoculated on the PDA plate with cellophane paper. Glass slides were placed on the PDA plate about 1 cm from the inoculated hyphae. The hyphae were cultured at 25°C for 3 days and grew to the middle of the cover slides. The glass slides with fresh hyphae were used to detect the viral proteins fused with GFP under 488 nm fluorescence and 100 × magnification.

To locate the p29 and p48 in the fungal cells, we fused the viral protein with the green fluorescent protein (GFP). As shown in Figure 7, p29 and p48 fusion proteins could be observed in the mycelium, but were hard to locate in specific organelles. p29 has been shown to be an important factor to suppress host cell RNA silencing and co-fractionates with trans-Golgi network membranes (Jacob-Wilk et al., 2006; Segers et al., 2006; Sun et al., 2006). Both p29 and p48 have been shown to be essential for fungal virulence, conidiation, and viral dsRNA accumulation; furthermore, p48 functions to initiate viral RNA replication (Deng and Nuss, 2008; Jensen and Nuss, 2014). The multifunction of these proteins justifies their existence in more than one organelle.

### Mitochondrial dysfunction upon hypovirus infection

The mitochondrion is the center of energy metabolism in eukaryotic cells and plays an important physiological role in the life process. Similar to any other virus, hypovirus needs to modulate mitochondrial bioenergetics to obtain sufficient energy for its replication. The virus may optimize the efficiency of mitochondrial respiration and ROS production to maximize benefits for the virus and lower the metabolic energy threshold for the host cell (El-Bacha and Da Poian, 2013).

The dysfunction of mitochondria has been implicated in several serious human diseases (Picone et al., 2014; Heo et al., 2017; Wang and Wei, 2017). In *C. parasitica*, a mutation in mitochondrion has been found to result in hypovirulence (Bertrand, 2000; Nuss, 2005). More interestingly, several mitochondrial proteins confirmed as virulence factors in previous studies, such as delta-1-pyrroline-5-carboxylate dehydrogenase (P5Cdh), proline dehydrogenase (Prodh), s-adenosylmethionine synthase (SAMS), and small GTPase Rho3 protein. P5CDH and ProDH, which are involved in glutamate biosynthesis and are required for virulence and mitochondrial integrity (He and DiMario, 2011; Yao et al., 2013; Rizzi et al., 2015), were found to be directly regulated by hypovirus (Table 3). SAMS took part in the methylation pathway and was important for virulence and transposition (Joardar et al., 2005; Liao et al., 2012). As a member of small GTPase family proteins, Rho3 was required in fungal growth, conidiation, and virulence (An et al., 2015). The chaos of energy metabolism would likely result in mitochondrial dysfunction that may impact the physiological activity of fungal cells.

## Conclusion

**Figure 8:**
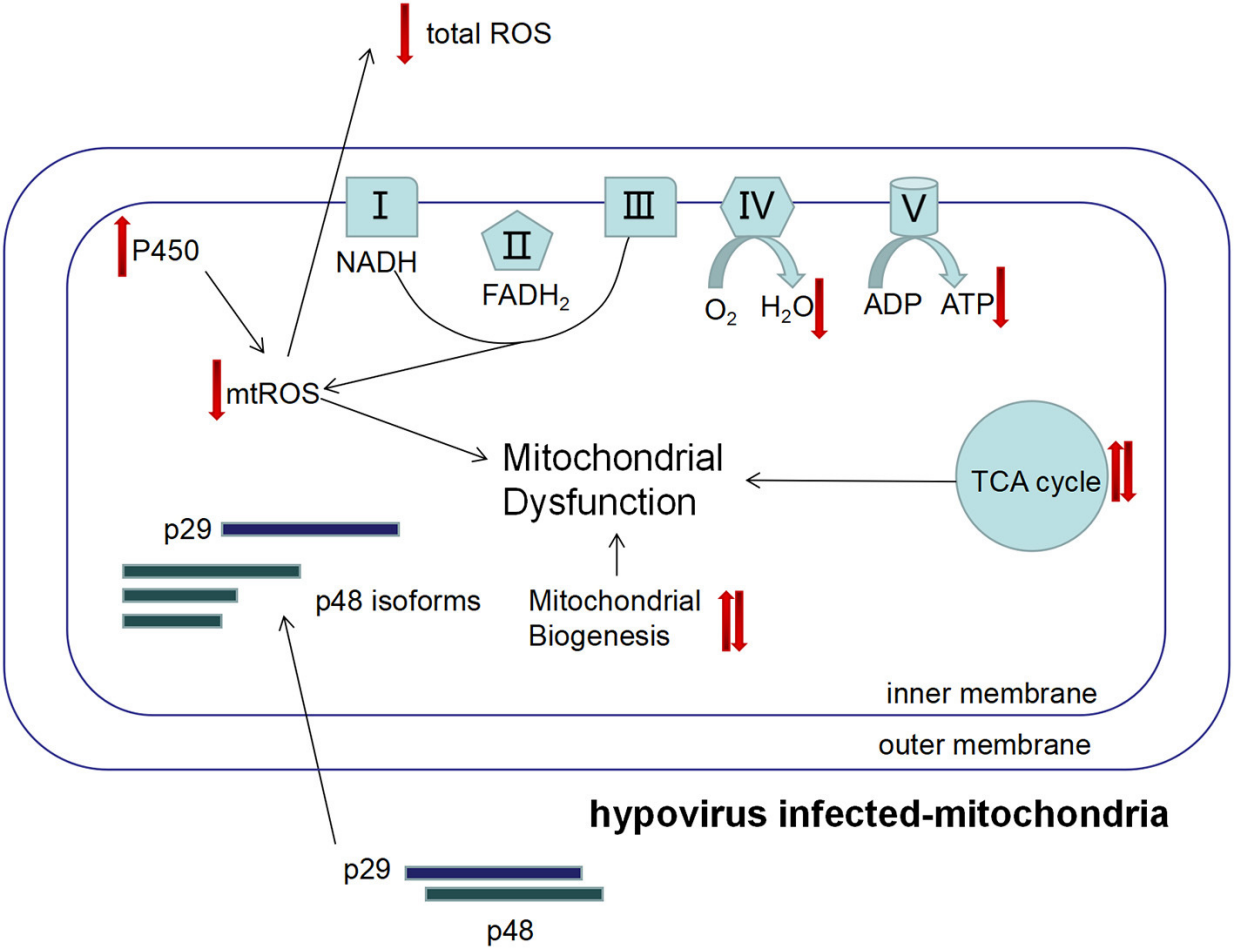
The proposed mechanism of viral perturbation to fungal mitochondrial function. The viral proteins p29 and p48 are transported into host mitochondria to induce proliferation of the host mitochondria that results in the increase in mitochondria. However, the mitochondrial unit capacity of the mitochondrion is lowered and function is impaired, e.g., the mtROS level is lower and the energy level is higher. Isoforms of p48 may play a role in these processes.

Hypovirus infection increased the total number of mitochondria, lowered the general ROS level, but elevated mitochondrial respiratory rate, and accelerated senescence of the mitochondria. Incongruousness in metabolism and signal transduction in mitochondria may result in dysfunction of the mitochondrion (Figure 8). In hypovirus-infected cells, decreased ROS level could be a protective mechanism to prevent the cells from damage caused by overloaded energy production in the mitochondrion.

## Data availability statement

The original contributions presented in the study are included in the article/Supplementary material, further inquiries can be directed to the corresponding authors. The data presented in the study are deposited in to the ProteomeXchange Consortium via the PRIDE partner repository, accession number PXD041756.

## Author contributions

JW, RL, and BC conceived and designed the experiments. RL and BC supervised the project and wrote the manuscript. JW and RQ wrote the manuscript draft. JW, XH, QF, and LS analyzed the experimental data. JW, RQ, ST, LZ, and SL performed the experiments. All authors contributed to the article and approved the submitted version.

## Supplementary material

Supplementary Table 1 The list of identified proteins by LC-MS/MS analysis of TMT-labeled mitochondrial preparations.
